# Irreducible Femur Head Fracture-Dislocation Treatment With Kocher-Langenbeck Approach With Flip Trochanteric Osteotomy: A Novel Approach

**DOI:** 10.7759/cureus.11969

**Published:** 2020-12-08

**Authors:** Rajesh Rana, Deepak Verma, Sudarsan Behera, Himansu Behera, Binod Raulo

**Affiliations:** 1 Orthopaedics, Institute of Medical Sciences and SUM Hospital, Bhubaneswar, IND; 2 Orthopaedics, All India Institute of Medical Sciences, Bhubaneswar, IND; 3 Orthopedics and Traumatology, Institute of Medical Sciences and SUM Hospital, Bhubaneswar, IND; 4 Orthopedics, Institute of Medical Sciences and SUM Hospital, Bhubaneswar, IND

**Keywords:** femoral head fracture-dislocation, avascular necrosis of head, pipkin classification, trochanteric flip osteotomy, femoral neck fracture, irreducible

## Abstract

Femoral head fracture-dislocations are rare, and irreducible cases are even less frequent. Truly irreducible fracture-dislocations must be differentiated from incomplete reduction due to incarcerated bone or soft tissue interposition. The Pipkin classification is commonly used to classify femoral head fractures. An urgent reduction is required in traumatic hip dislocations to reduce the risk of avascular necrosis (AVN) of the femoral head. However, in femoral head fractures, the dislocated hip cannot be reduced easily due to incarcerated bone or soft tissue. In an irreducible fracture hip dislocation, It is not advisable to attempt to reduce it repeatedly because sometimes femoral head fracture-dislocation is associated with the impacted fracture of the femoral neck. It may lead to iatrogenic femoral neck fracture. Hence, in such cases, immediate open reduction and internal fixation are recommended. The Kocher-Langenbeck approach can be used for reduction and safe surgical dislocation with flip trochanteric osteotomy for fixation as a novel approach.

## Introduction

The incidence of femoral head fracture-dislocation is between 8-26% [1]. Hence, it is clear that femoral head fracture-dislocation is a rare condition [2], and dislocations that cannot be reduced by the close method is even rarer [3,4]. The most common cause is high-energy road traffic accidents with associated injuries of the ipsilateral knee, acetabulum, femoral neck, sciatic nerve, and pelvis. Usually, the position of the head and hip during axial loading determines the type of injury [5]. Early diagnosis and emergent reduction are needed to prevent future complications like avascular necrosis (AVN) of the head and arthritis [6]. The Pipkin classification is commonly used in these cases [7]. Necessary imaging procedures like X-ray and CT scan should be done before the reduction attempt as there is always an increased risk of the iatrogenic neck of femur fracture after a closed reduction [8]. True irreducible fracture-dislocations are where the femoral head cannot be reinserted into the acetabulum, and they must be differentiated from incomplete reduction caused by the femoral head fragment or soft tissue interposition. It remains controversial as to which treatment approach should be used in such cases [9]. In our case, we used the extended Kocher-Langenbeck approach with a flip trochanteric osteotomy to treat the irreducible femoral head fracture-dislocation.

## Materials and methods

From January 2018 to January 2020, we encountered 30 cases of fracture of the head of the femur with dislocation of the hip. Among those, six cases had a failed reduction history. We tried closed reduction in the usual manner both without anaesthesia and then under anaesthesia. All six fractures were then reduced by the open surgical method. Pathological fractures, anterior dislocations, and associated acetabulum fracture cases were excluded.

**Figure 1:**
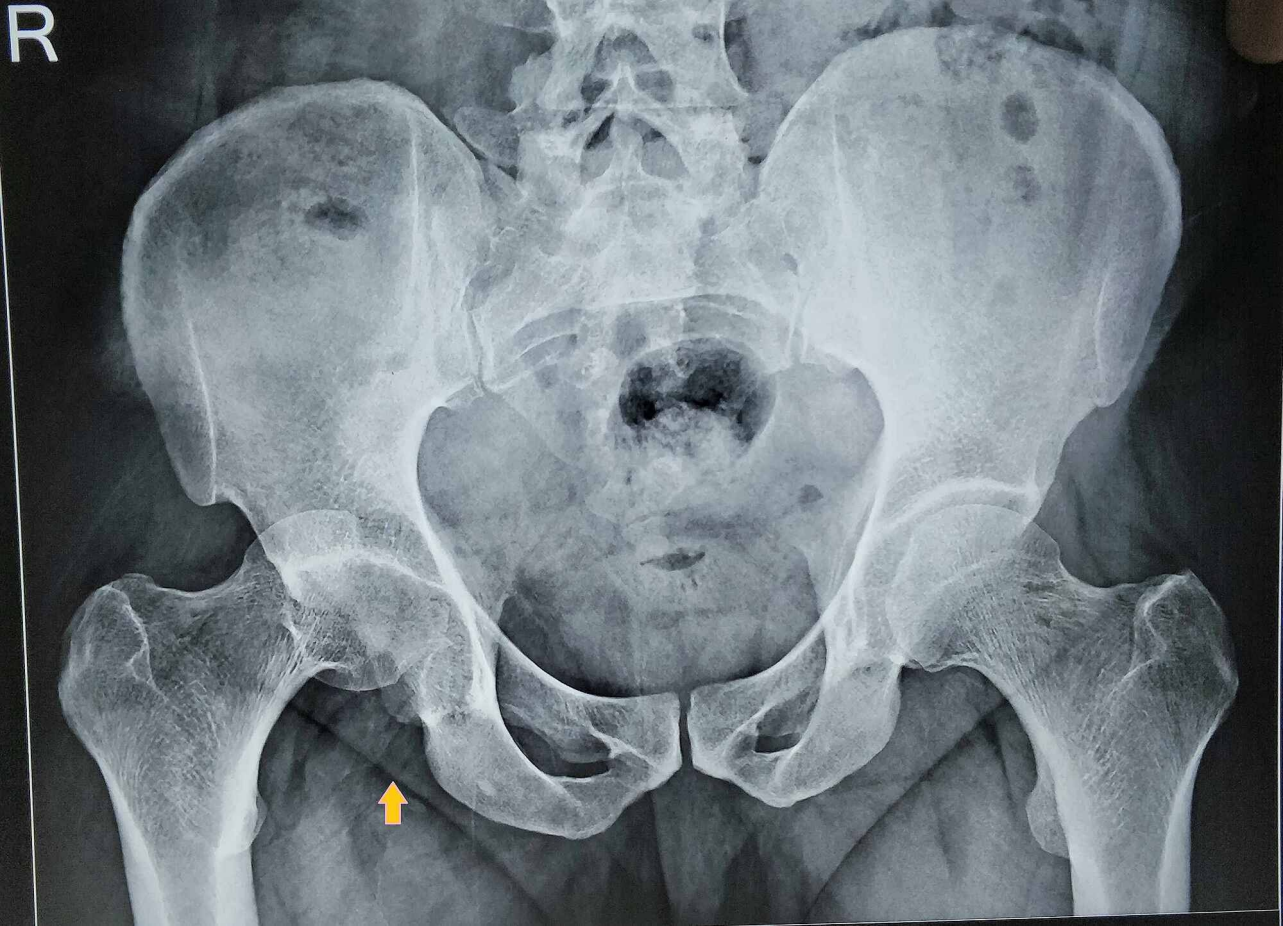
X-ray showing Pipkin fracture-dislocation (arrow)

There were four males and two females among the six patients. The average age of the patients was 42 years (range: 32-54 years). Five patients had been injured by road traffic accidents, and one patient had fallen from height. All patients were evaluated with a physical examination, anterior- and posterior-view radiographs of both hips, and CT scans with 3D reconstruction. On physical examination, all patients were found to have a slight internal rotation of the leg with shortening and slight flexion. The globular head was palpable in the gluteal region pointing towards dislocation of the hip. All range of movements were painful. All patients underwent anterior-posterior (AP) radiography in the preoperative and postoperative periods. X-rays were repeated at repeated follow-up consultations every two months for one year, followed by consultations every six months as a routine. All preoperative X-rays showed fractured head fragments in the acetabulum, and the proximal head fragment with continuity with neck-shaft dislocated posterior-superiorly. The proximal head fragment was tightly hinged at the posterosuperior lip of the acetabulum and lateral iliac cortical bone (Figure 1).

**Figure 2:**
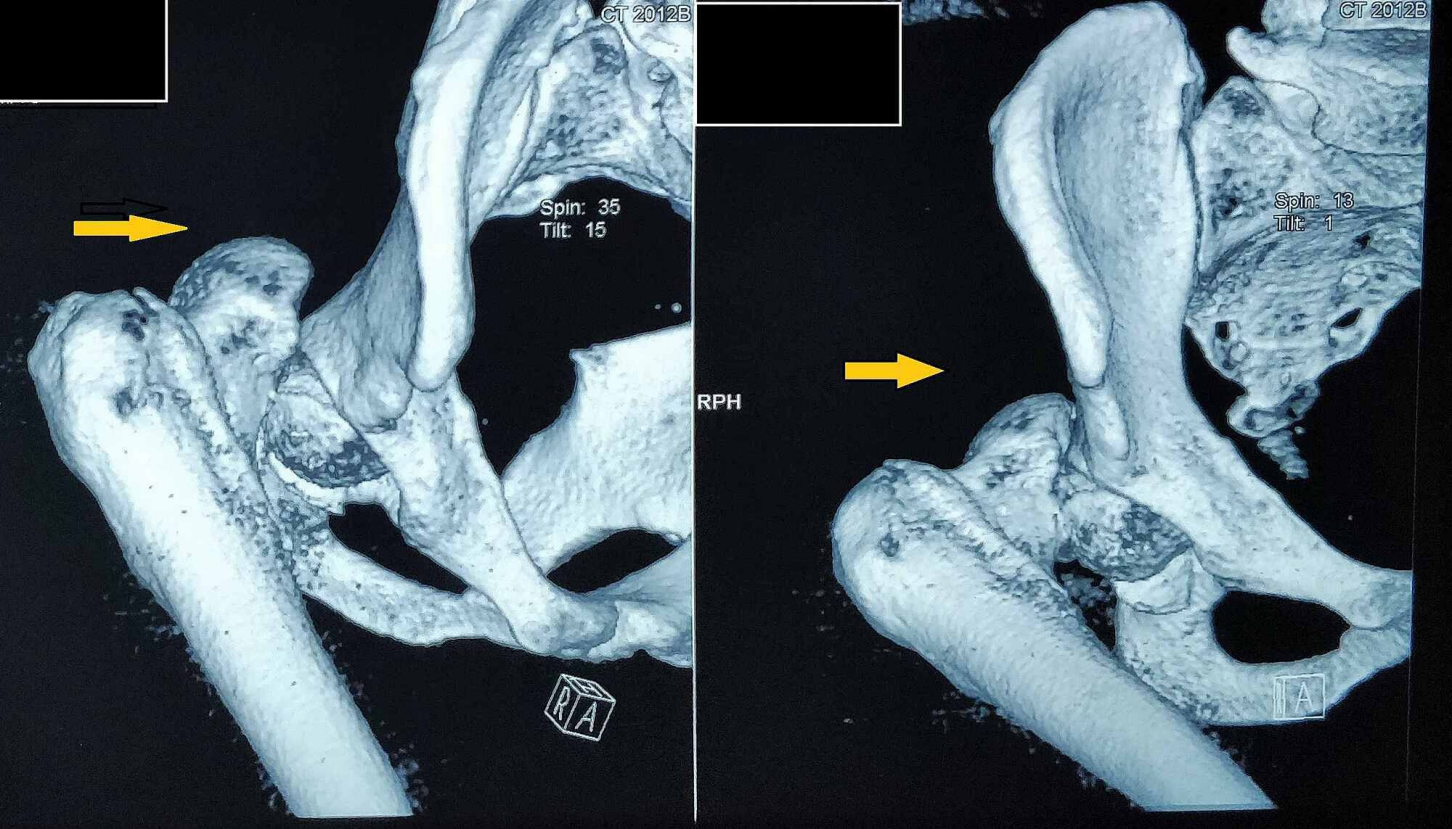
CT scans showing Pipkin fracture-dislocation (arrows) CT: computed tomography

All patients underwent CT scans with coronal and sagittal sections with 3D reconstructions. CT scans showed the details in a better way with fragment locations and sizes (Figure 2).

After the confirmation of diagnosis, we tried close reduction in all cases within six hours of injury. We tried a minimum of two attempts (range: one to four) at close reductions in all cases before surgery. All patients were taken to the operation theatre for reduction surgery within a mean time of 12.5 hours (range: 6-24 hours) from the time of the injury. One patient had other abdominal injuries, which took more time to stabilize before surgery could begin.

Surgical technique and technique of reduction

Once the patients were deemed medically fit for surgery, they were shifted to the operation theatre. All patients were given general anaesthesia and adequate muscle relaxation. We positioned patients in the lateral position with padded support at the presacral and pubic symphysis area. The anticipated surgical site was shaved and cleaned with betadine scrub. The entire injured lower limb, including the hip, was draped and was kept free for intraoperative manipulation. We prepared the surgical site with iodine solution followed by an isopropyl alcohol solution. The patients received tranexamic injections and 1.5 gm cefuroxime injections during induction. We reduced the dislocated hip through the Kocher-Langenbeck approach. The fixation of the Pipkin fracture was planned by flip trochanteric osteotomy and surgical safe dislocation, which only minimally damages the vascular supply to the head.

**Figure 3:**
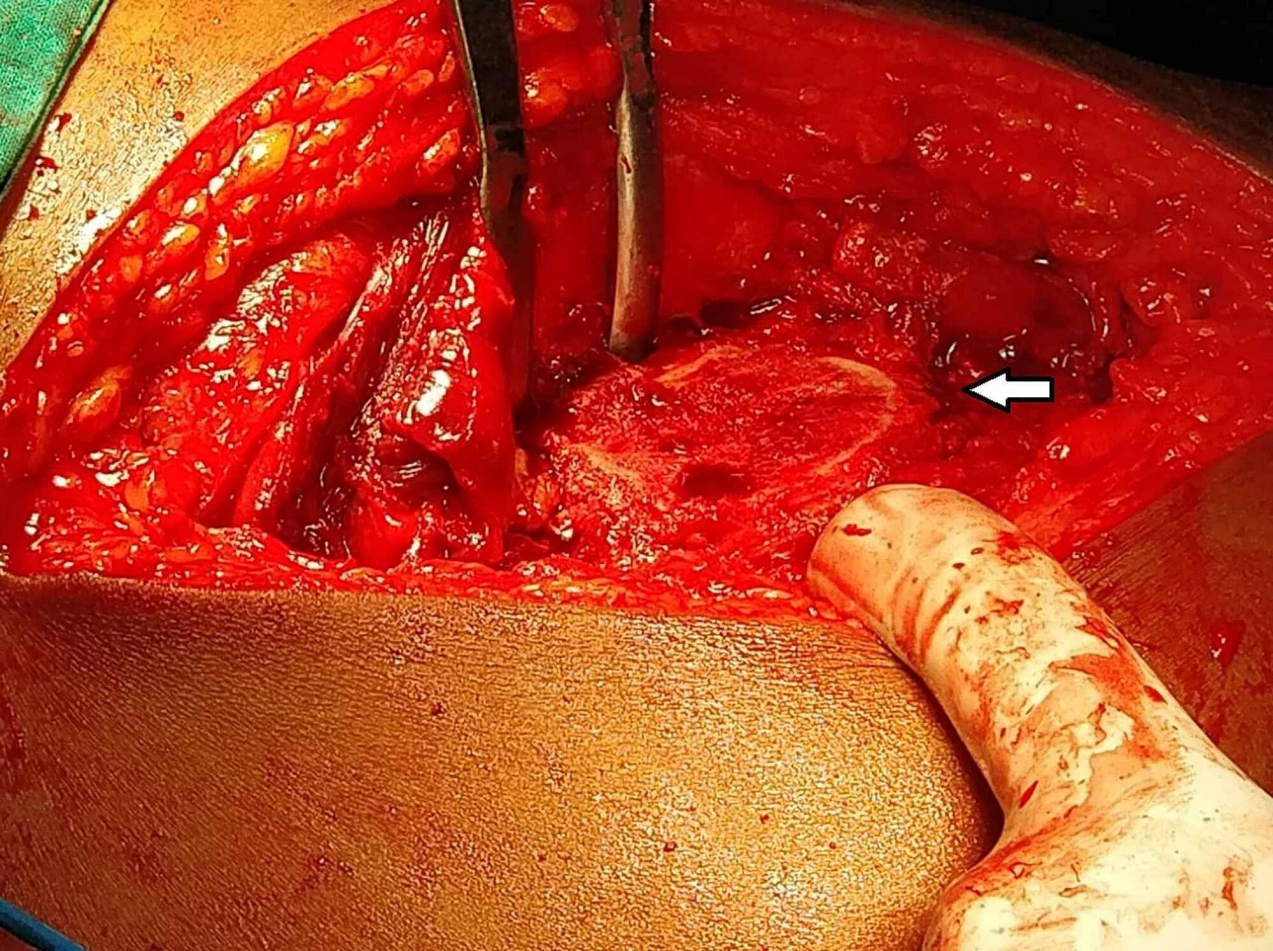
Flip trochanteric osteotomy

For osteotomy, the leg was internally rotated. From the posterior border of the vastus lateralis, origin 1.5 cm of the greater trochanter was osteotomised. The osteotomised fragment had gluteus medius attachment above and vastus lateralis below. The fragment was displaced anteriorly (Figure 3).

**Figure 4:**
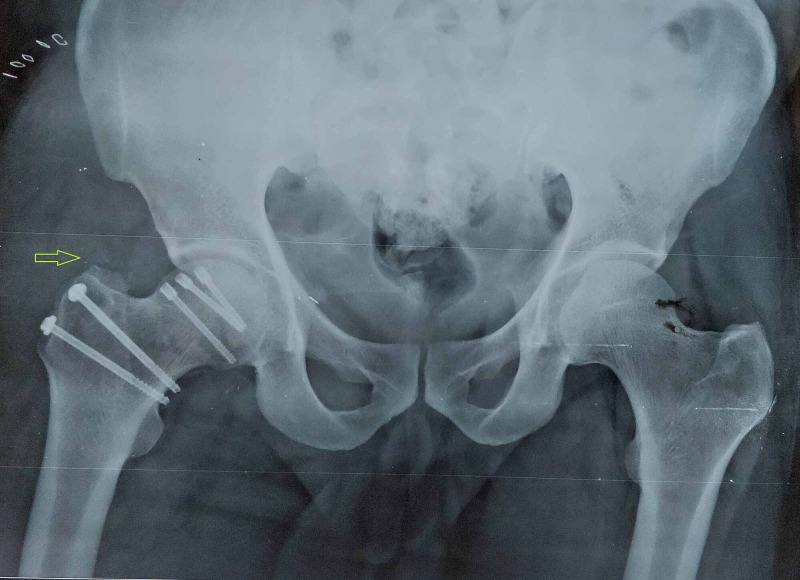
Postoperative X-ray

The capsule was incised with a Z-shaped incision. We dislocated the hip by flexion and external rotation of the leg. The head fragment was taken out from the acetabulum by cutting ligamentum teres. The joint cavity was inspected, and complete lavage was done to remove bone debris. Femur head fractures were fixed with Herbert screws (headless screws), and the hip joint was reduced (Figure 4).

The capsule and labral complex were repaired meticulously. Soft tissue layers were closed. Trochanteric osteotomy fragment was fixed with 4 mm cancellous screws. The patients were kept non-weight-bearing for the first six weeks, but all movements of the hip were allowed. After six weeks, partial weight-bearing was allowed with a walker. All fractures united within four to six months, and patients were allowed full weight-bearing.

## Results

Total follow-up periods ranged from eight to 18 months, with an average follow-up period of 10.8 months. The average fracture union time was three to four months. One patient had the perioperative complication of deep vein thrombosis (DVT). DVT was treated with proper thrombolytic and it subsided without any further complications. At the six-month follow-up, two patients showed signs and symptoms of AVN of the head. Both the patients had had their operations delayed by more than eight hours. The average intraoperative time had been 1.5 hours. Two patients had other fractures like humerus fracture and tibial spine avulsion fracture. Both these cases had taken more intraoperative time because of simultaneous fixation. Average blood loss had been 450 ml. In four of the cases, the proximal femur had dislocated through bone and labral intervals. The tough labral tissue had been blocking reduction. After the release of labral tissue, the reduction had been made possible. In two cases, the fractured femoral head had been hinged at the superolateral margin of the acetabulum, which had been preventing reduction. The mean Harris hip score at the six-month follow-up was 86 with excellent results in three cases (50%), a good result in one case (16%), and fair results in two cases (33%) cases. Fair results were due to the initial symptoms of AVN head. Otherwise, all patients had good functional outcomes at their six-month follow-ups. All patients were evaluated with a postoperative radiograph, and further radiographs at each subsequent follow-up.

## Discussion

Irreducible posterior dislocation of the hip is always an indication for open reduction. Only 2-15% of dislocated hips are irreducible by closed reduction methods. Irreducible fracture-dislocation of femoral head without a fracture of the acetabulum constitutes about 10% of femoral head fracture-dislocation [4]. Commonly in such types of cases, the proximal femur herniates through the superior-posterior gap between the acetabular rim and the labrum [10,11]. Other causes of irreducibility are interposition of pyriformis, ligamentum teres, labrum, and, sometimes, buttonholing through the posterior capsule [12,13]. Sometimes, the rotation of the head fragment around the ligamentum teres and osteochondral fragment prevent reduction. In some of our cases, the rotation of the fragment was the main culprit in preventing reduction. In a few cases, the proximal head fragment gets hinged at the superior poster wall of the acetabulum. Often, a fractured neck of the femur is associated with a femoral head fracture or get the neck of femur fracture while vigorous close reduction attempt. This should be diagnosed before any attempt at reduction. Diagnosis is always via plain radiograph along with a CT scan. CT scan always gives a detailed idea about fragment sizes and other predisposing causes for irreducibility.

The fractured head of the femur with dislocation is always a surgical emergency, and irreducible cases should be fixed with open reduction and internal fixation. Delay in reduction increases the chance of osteonecrosis of the head [14,15]. The chance of AVN is up to 14.8% when it is reduced after 12 hours [16]. Studies show that in dislocation of the hip, the extraosseous blood supply is disrupted but intraosseous blood supply is maintained [17]. The choice of an appropriate approach is always a dilemma. In such a situation, irreducible dislocation of the head can be reduced first with open reduction. Flip trochanteric osteotomy with a Ganz safe surgical dislocation can be a novel approach for the fixation of such types of fracture [18,19]. This approach gives excellent intraarticular access without hampering blood supply to the head. Intraarticular fragments can be removed in a much better manner. Henle et al. have reported in their study that this approach had good to excellent outcomes in comparison to other approaches like the Watson-Jones approach, Smith-Petersen approach, and Kocher-Langenbeck approach [20]. Capsulolabral tears should be repaired meticulously and, if required, should be fixed with suture anchors. Capsule and labrum repairs have a favorable prognostic factor with increased stability and even distribution of load.

## Conclusions

Irreducible femoral fracture-dislocations can be reduced and fixed effectively with a Kocher-Langenbeck approach using flip trochanteric osteotomy. This approach provides direct antero-inferomedial and dorsal access to the fracture site because of musculocapsular lesions caused by posterior dislocation. It causes less soft tissue trauma. The blood supply to the head of the femur is preserved, and it is associated with good outcomes.
